# Resilience or vulnerability? thalamic subdivision connectivity in trauma-exposed individuals: a 7T resting-state fMRI study

**DOI:** 10.1038/s41398-025-03774-w

**Published:** 2025-12-13

**Authors:** Nibal Khudeish, Rajkumar Ravichandran, Abdulrahman S. Sawalma, Raghad Kiwan, Shukti Ramkiran, Jana Hagen, N. Jon Shah, Irene Neuner

**Affiliations:** 1 Institute of Neuroscience and Medicine, Institute of Neuroscience and Medicine (INM-4), Forschungszentrum Jülich GmbH, Jülich, Germany; 2 Department of Psychiatry, Psychotherapy and Psychosomatics, RWTH Aachen University, Aachen, Germany; 3 Jülich Aachen Research Alliance - Brain (JARA – BRAIN) – Translational Medicine, Aachen, Germany; 4 Institute of Neuroscience and Medicine, Institute of Neuroscience and Medicine (INM-11), Forschungszentrum Jülich GmbH, Jülich, Germany; 5 Department of Neurology, Rheinisch-Westfälische Technische Hochschule Aachen (RWTH) Aachen University, Aachen, Germany

**Keywords:** Neuroscience, Psychiatric disorders

## Abstract

Why some individuals are resilient to trauma while others develop psychopathology remains a baffling question in mental health research. Trauma-related conditions like post-traumatic stress disorder (PTSD), major depressive disorder (MDD), and anxiety disorders affect millions worldwide, emphasizing the need to understand the neural mechanisms that underlie these divergent outcomes. Through the use of ultra-high field (UHF) 7 T imaging, this study sought to investigate how thalamic functional connectivity differentiates resilience from vulnerability in trauma-exposed individuals. To that end, UHF 7 T resting-state functional magnetic resonance imaging (rs-fMRI) was applied to a group of 46 refugees from the Levant region, including 23 symptomatic (PTSD, MDD, or anxiety disorders) and 23 asymptomatic participants. Using the CONN toolbox, we conducted seed-to-voxel analyses focused on the thalamic subregions defined by the Human Brainnetome Atlas. Our results revealed significant connectivity alterations in the right medial prefrontal thalamus (mPFtha), the lateral prefrontal thalamus (lPFtha), and the occipital thalamus (Otha). Symptomatic individuals exhibited hyperconnectivity between the thalamic subregions and the somatosensory, visual, and cerebellar networks, along with reduced inter-thalamic connectivity, suggesting emotional dysregulation and hypervigilance. In contrast, asymptomatic participants displayed increased inter-thalamic connectivity and hypoconnectivity with these networks, reflecting efficient sensory integration and emotion regulation. Reduced inter-thalamic connectivity was found to correlate with lower resilience, underscoring the importance of effective thalamic communication for emotional stability. Taken together, our findings suggest that thalamic dysregulation contributes to vulnerability, while increased inter-thalamic connectivity fosters resilience through better sensory and emotion regulation. Thus, this study affords valuable insights into potential neural targets for interventions, which may help enhance resilience in trauma-exposed populations.

## Introduction

Exposure to trauma is a significant public health challenge with profound psychological consequences. It is estimated that approximately 70% of adults worldwide experience at least one traumatic event during their lifetime, often facing multiple exposures [1], which increase the likelihood of developing various psychological disorders, including post-traumatic stress disorder (PTSD), major depressive disorder (MDD), and anxiety disorders [2, 3]. In populations affected by conflict and war, the prevalence of these disorders is notably high [4]. While PTSD is frequently studied, it is not the only psychiatric disorder to affect such populations. Depression, anxiety, and suicidality often coexist with or occur independently of PTSD [5, 6]. The cumulative impact of war, persecution, and the ongoing stressors associated with displacement, put individuals from conflict zones at high risks of overlapping and independent psychopathologies, emphasizing the critical need for comprehensive mental health interventions in these vulnerable populations.

Despite the widespread impact of trauma, individuals exhibit considerable variability in their psychological responses. Some develop debilitating symptoms, while others demonstrate resilience, maintaining or quickly recovering mental health despite adversity [7]. In this context, resilience is characterized by an individual’s ability to “bounce back” after exposure to stressors, with factors such as inner strength, competence, optimism, flexibility, and effective coping mechanisms playing key roles [8, 9]. However, the neurobiological mechanisms that underlie resilience, particularly among individuals who remain asymptomatic despite trauma exposure, remain insufficiently understood. Investigating these mechanisms is crucial for designing interventions that may enhance resilience in trauma-affected populations.

Resilience arises from a dynamic interplay between biological and psychological factors, enabling individuals to adapt to adversity [10, 11]. Neurobiologically, it is believed to involve adaptive changes in brain function and connectivity, particularly in regions responsible for emotion regulation, sensory processing, and stress response [10, 12, 13]. Among these regions, the thalamus, a central hub for sensory processing and emotion regulation, plays a critical role in shaping how individuals perceive and react to trauma [14, 15], as it is involved in the integration of sensory input, regulation of attentional processes, and modulation of emotional responses to external stimuli [16]. Additionally, its role in coordinating the communication between higher-order cognitive processes and autonomic functions makes it essential for stress response and the maintenance of emotional balance [17]. Alterations in thalamic connectivity have been implicated in both the development of trauma-related psychopathologies and resilience, suggesting that thalamic networks may be key to understanding individual differences in trauma response [15].

Most neuroimaging research on trauma-related psychopathology has concentrated on the amygdala, hippocampus, and medial prefrontal cortex, given their well-established roles in emotional processing and stress regulation [18–20]. In contrast, the thalamus has received comparatively less attention, despite its central function in arousal regulation, salience detection, and sensory integration [21, 22]. Emerging evidence increasingly highlights the importance of thalamic connectivity in trauma-related conditions, including PTSD, MDD, and anxiety disorders [23, 24]. Altered thalamocortical connectivity patterns, particularly with cortical regions involved in emotion regulation such as the medial prefrontal cortex and insula, have been linked to overlapping symptom dimensions across these disorders [25–27]. Collectively, these findings suggest that alterations in thalamocortical connectivity patterns may represent a shared neurobiological substrate that influences both vulnerability and resilience in trauma-exposed individuals, offering a promising avenue for mechanistic investigation and the development of targeted interventions.

Advanced neuroimaging techniques are essential for exploring these connectivity patterns, particularly in understanding the neurobiological mechanisms of resilience and vulnerability. Resting-state functional magnetic resonance imaging (rs-fMRI), using ultra-high field (UHF) scanners, is an advanced tool for mapping neural circuits with a high spatial resolution. Studies have demonstrated that higher magnetic field strengths, such as 7 T and beyond, offer increased sensitivity, allowing for the precise detection of subtle neural signals and small-scale connectivity changes, which may be otherwise missed [28, 29]. Thus, this technology enables a detailed examination of thalamic connectivity, which is crucial for understanding the nuanced differences between resilient individuals and those who develop trauma-related psychopathology [30]. The advantages of UHF MRI, including higher signal-to-noise ratio and enhanced spatial resolution, have proved useful in analyzing brain connectivity and detecting critical network alterations implicated in psychiatric disorders [31, 32]. By providing a more comprehensive view of brain networks, UHF rs-fMRI enhances our understanding of the neurobiological foundations of resilience and offers insights into potential therapeutic targets.

In our study, we employed 7 T UHF rs-fMRI to investigate thalamic connectivity in a trauma-exposed population of refugees categorized on the basis of symptom profiles. Building on prior research that underscores the role of altered thalamic connectivity in trauma-related disorders [15, 33, 34], we conducted an exploratory seed-to-voxel analysis using a multiple regression model. This approach aims to investigate functional connectivity differences between resilient (asymptomatic) and vulnerable (symptomatic) individuals. We hypothesized that asymptomatic individuals would exhibit increased inter-thalamic connectivity, supporting more efficient sensory integration and emotion regulation.

In addition, based on previous findings of altered sensory processing in trauma-related disorders [24, 35–38] and recent evidence implicating thalamo-somatosensory/parietal connectivity as a marker of general psychiatric vulnerability [26], we hypothesized that resilient individuals would exhibit reduced functional connectivity between the thalamus and regions associated with sensory-motor functions. By analyzing thalamic connectivity at the level of specific subregions, this study sought to advance the understanding of the neurobiological underpinnings of trauma responses and resilience, potentially identifying novel neural markers that might inform future therapeutic interventions.

## Methods

### Participants

Forty-six right-handed refugees from the Levant region (mean age: 27.8 ± 8.2 years; 13 females, 33 males) were recruited via flyers and snowball sampling. Inclusion criteria were MRI compatibility, origin from the Levant region, right-handedness, absence of neurological disorders, and no current substance dependence or addiction. Sample size was determined a priori using G*Power 3.1 for a general linear model (fixed model, R² increase) with two tested predictors and two covariates (total predictors = 4), assuming a large effect size (f² = 0.35), α = 0.05, and 1-β = 0.85. This analysis indicated a minimum total sample of 35 participants [39]. Our final sample of 46 participants exceeded this threshold and aligns with previous neuroimaging studies in trauma-exposed populations [40, 41]. All assessments were conducted in Arabic, the participants’ native language, to minimize linguistic bias and enhance response accuracy. Participants first completed a battery of questionnaires, including the Resilience Scale (RS-25) [42], the Beck Depression Inventory (BDI) [43], the Harvard Trauma Questionnaire (HTQ) [44], and a trauma exposure checklist. This was followed by a structured interview conducted by two well-trained researchers using the Mini International Neuropsychiatric Interview (MINI) to ensure consistency across assessments [45]. Although the MINI is not a substitute for a comprehensive clinical assessment, it is a structured and validated interview commonly used in research to screen for and categorize psychiatric disorders based on DSM criteria. In cases where PTSD symptoms were indicated, the Clinician-Administered PTSD Scale (CAPS) was administered to confirm PTSD status [46], and HTQ cutoffs were used to provide additional validation.

Participants were then categorized into two groups based on this multi-step assessment: a symptomatic group (n = 23; mean age: 30.3 ± 10.2 years; 7 females, 16 males), which included individuals meeting research-based diagnostic criteria for PTSD, major depressive disorder (MDD), or anxiety disorders (e.g., social anxiety disorder, panic disorder, or generalized anxiety disorder); and an asymptomatic group (n = 23; mean age: 25.2 ± 4.8 years; 6 females, 17 males), consisting of participants without any current or past psychiatric diagnosis. Group assignment was based solely on clinical interview outcomes and not on resilience scores. The RS-25 was administered to all participants to assess resilience dimensionally, and it was used exclusively for post hoc correlation analyses to examine individual variability across groups. None of the participants was taking psychiatric medication at the time of the study. The study adhered to the Declaration of Helsinki and was approved by the Ethics Committee of the Faculty of Medicine at RWTH Aachen University. All participants provided written informed consent prior to participation.

### Psychological measures

The **Harvard Trauma Questionnaire (HTQ)** was utilized to assess trauma-related symptoms among participants. This version has been clinically validated for Arabic-speaking refugee populations and is widely used in cross-cultural research involving trauma survivors. In this study, both the HTQ PTSD subscale (Part III), which evaluates core post-traumatic stress symptoms based on DSM criteria, and the HTQ total score, which includes additional items related to somatic and depressive symptoms (Part IV), were analyzed. Items are rated on a Likert scale, capturing the frequency and severity of symptoms such as detachment, concentration difficulties, and sleep disturbances [44].

The **Resilience Scale (RS-25)**, also accessed in Arabic, was used to measure psychological resilience, which is defined as the capacity to recover from adversity. This scale includes 25 items, rated on a 7-point Likert scale ranging from “1 = strongly disagree” to “7 = strongly agree.” The RS-25 evaluates personal competence, acceptance of self and life, and the ability to maintain a positive outlook [42]. The Arabic version of this scale has been validated for use in Arabic-speaking populations.

The **Beck Depression Inventory (BDI-II)** was used to measure the severity of depressive symptoms. The Arabic version of this inventory includes 21 items assessing aspects such as mood, pessimism, and suicidal thoughts. Each item is rated from 0–3, with higher scores indicating more severe symptoms. This version has been validated for Arabic-speaking individuals, ensuring its accuracy in capturing depressive symptoms in the study [43, 47].

### Data acquisition

MRI data were acquired using a 7 T MAGNETOM Terra scanner (Siemens Healthineers, Erlangen, Germany) equipped with a 1Tx/32Rx head coil (Nova Medical, Wilmington, MA, USA) and located at Forschungszentrum Jülich.

The rs-fMRI data were collected using a 2D T2* weighted multiband accelerated echo-planar imaging (EPI) protocol developed by the Center for Magnetic Resonance Research (CMRR) at the University of Minnesota, USA [48]. Imaging parameters included a repetition time (TR) of 2000 ms, an echo time (TE) of 25 ms, a flip angle (FA) of 70°, and a multiband acceleration factor of 4. The resulting images had an isotropic resolution of 1.3 mm, a field of view (FOV) of 220 × 220 mm², and a matrix size of 168 × 168. A total of 305 volumes, each containing 100 slices, were recorded. Participants were instructed to keep their eyes closed, and avoid falling asleep. The scanning environment was kept dark with the lights switched off. To generate the fieldmaps necessary for correcting susceptibility distortions caused by B0 field inhomogeneities, a “blip-up blip-down” acquisition technique was applied [49]. For this, the protocol was slightly adjusted by flipping the phase encoding direction by 180°, followed by the acquisition of two additional volumes.

For structural imaging, a 3D T1-weighted magnetization-prepared rapid gradient-echo (MP2RAGE) sequence was used. Two inversion images (INV1 and INV2) were acquired at different inversion times (TI) and flip angles (FA). INV1 had a TI of 840 ms and FA of 5°, while INV2 had a TI of 2370 ms and FA of 6°. These images were combined to produce a high-resolution structural image corrected for bias, proton density, and T2* contrast, achieving a resolution of 0.75 mm isotropic [50]. The FOV was 225 × 240 mm², with a matrix size of 300 × 320, covering 208 sagittal slices.

### Data preprocessing and denoising

The MRI data, initially stored in DICOM format, were converted to a NIfTI format using “dcm2niix,” which is part of the MRIcron software. The susceptibility-induced off-resonance field (fieldmap) was estimated using a method implemented in FSL [51], based on established approaches for correcting susceptibility distortions [49]. Subsequent preprocessing was performed using the CONN toolbox [52](v.22.a) [53] for SPM12 (v7771, Wellcome Department of Cognitive Neurology, University College London, UK; https://www.fil.ion.ucl.ac.uk/spm/) in MATLAB R2024a (Matworks Inc., Sherborn, MA, USA).

The preprocessing of the functional and anatomical data was performed using a predefined pipeline within the CONN toolbox for volume-based analysis, which included direct normalization to MNI space, with all preprocessing parameters set to their default values. Functional images underwent preprocessing to correct for head motion and susceptibility-induced distortions. First, fieldmaps were converted into voxel displacement maps, allowing for voxel-wise correction of susceptibility distortions. This was followed by realignment and unwarping using SPM’s realignment procedure to further correct motion-related artifacts. Then, the image center was set to the origin coordinates (0, 0, 0) to ensure accurate anatomical alignment [54].

Next, slice-timing correction was applied to adjust for differences in acquisition time between slices. Outlier scans were identified using Artifact Detection Tools (ART), with thresholds set at 0.9 mm for framewise displacement and five standard deviations for global signal changes [55]. Both functional and anatomical data were segmented into gray matter, white matter, and cerebrospinal fluid (CSF). After segmentation, the data were normalized directly into MNI152 standard space and downsampled to a 2 mm isotropic resolution for functional images [56]. A finer, 1 mm isotropic resolution was used for the anatomical data. Finally, functional images were smoothed using an 8 mm full-width at half maximum (FWHM) Gaussian kernel to reduce noise and enhance the signal-to-noise ratio.

Following preprocessing, denoising was conducted to remove confounding influences from the blood-oxygen-level-dependent BOLD signal. This involved the application of the anatomical component-based noise correction (aCompCor) method, which regressed out five principal components, each from the white matter and CSF signals [57]. Additional nuisance regressors included the six realignment parameters from motion correction, their first derivatives, and scrubbing regressors based on the detected outliers. Linear detrending and session effects were also accounted for to minimize transient BOLD signal fluctuations. Finally, a temporal band-pass filter was applied (0.008–0.09 Hz) to eliminate physiological noise, such as the one arising from cardiac and respiratory cycles.

### Seed-to-voxel connectivity analysis

#### First-level analysis

**Fig. 1:**
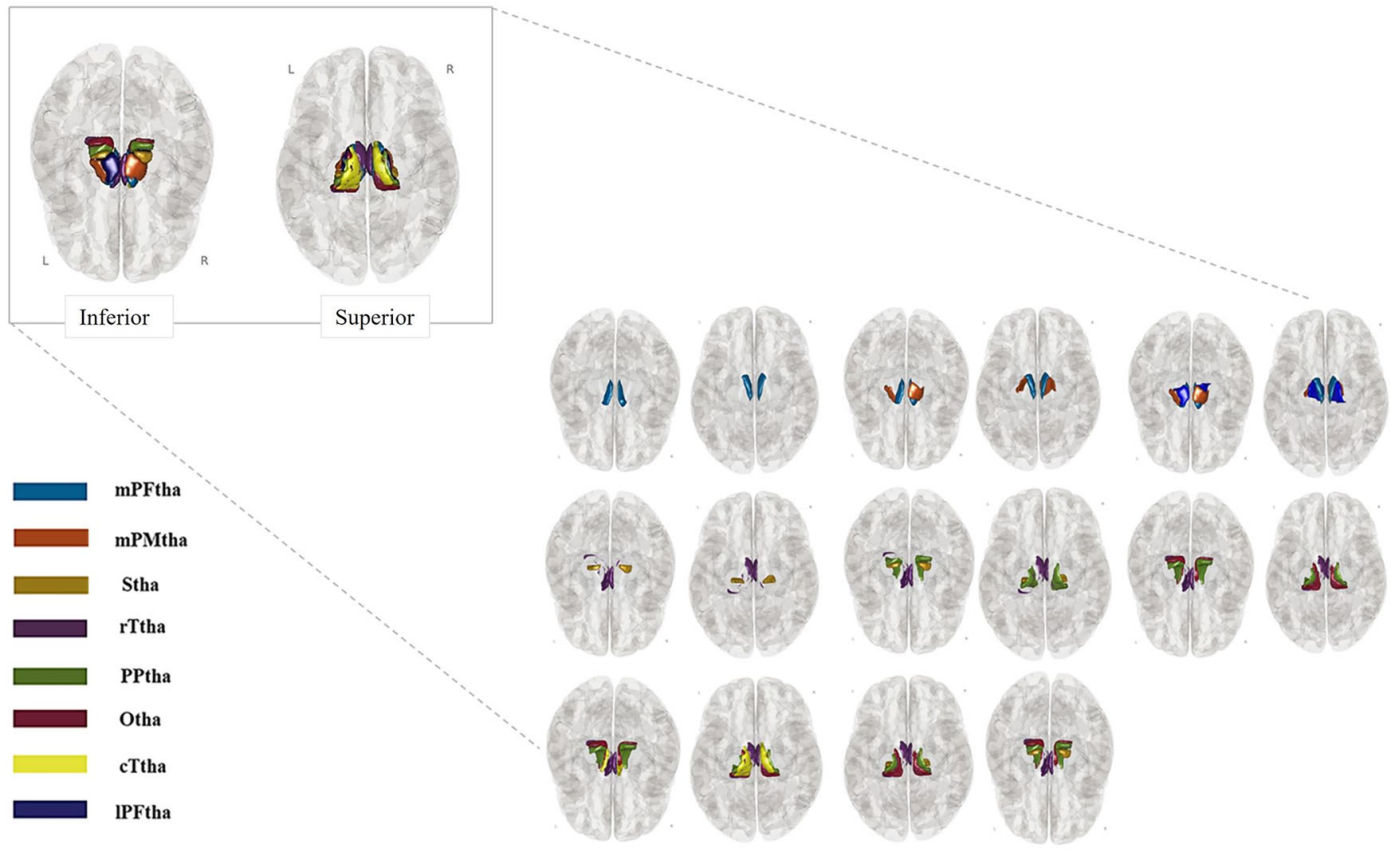
Thalamic subregions as defined by the Human Brainnetome Atlas. Glass brain displays represent the structural axial views (inferior and superior representations) illustrating the thalamic subregions used as seed regions (ROIs) in the seed-to-voxel analysis, as defined by the Human Brainnetome Atlas. The subregions include the medial prefrontal thalamus (mPFtha), premotor thalamus (mPMtha), sensory thalamus (Stha), rostral temporal thalamus (rTtha), posterior parietal thalamus (PPtha), occipital thalamus (Otha), caudal temporal thalamus (cTtha), and lateral prefrontal thalamus (lPFtha).

Functional connectivity analyses were conducted using the CONN toolbox [53]. Seed-to-voxel connectivity maps were generated by correlating the time series of seed regions with all other voxels in the brain. Based on the Human Brainnetome Atlas (HBA) [58], eight thalamic subregions were selected in each hemisphere, resulting in 16 regions of interest (ROIs) (Fig. 1). These subregions included the medial prefrontal thalamus (mPFtha), the premotor thalamus (mPMtha), the sensory thalamus (Stha), the rostral temporal thalamus (rTtha), the posterior parietal thalamus (PPtha), the occipital thalamus (Otha), the caudal temporal thalamus (cTtha), and the lateral prefrontal thalamus (lPFtha). Fisher-transformed bivariate correlation coefficients were calculated to represent the functional connectivity between seed regions and target areas using a weighted general linear model (weighted-GLM) [59], with scans weighted by a step function convolved with the SPM canonical hemodynamic response function to account for transient magnetization effects.

#### Group-level analysis

Functional connectivity differences between the Symptomatic and Asymptomatic groups were assessed using a General Linear Model (GLM) [59], with age and gender as covariates. A contrast vector [1 -1 0 0] was applied to test group differences while statistically controlling for age and gender. Voxel-level hypotheses were evaluated using multivariate parametric statistics, and random effects were modeled across subjects. Cluster-level inferences were based on Gaussian Random Field theory [60], using a voxel-level threshold of *p* < 0.001 and a cluster-level threshold of p < 0.05, corrected for multiple comparisons using false discovery rate (FDR) at the voxel level. To further control for multiple testing across the 16 seed-to-voxel analyses, FDR correction was also applied across seeds. These values are reported in Table 2 as *P-value (FDR-corrected across seeds)*.

### Statistical and correlation analysis

Group differences in demographic and clinical variables were assessed to verify comparability between the symptomatic and asymptomatic groups. Independent-samples t-tests were applied to continuous measures, including age and psychological scores (RS-25, BDI, HTQ-total, and HTQ-PTSD subscale). Gender distribution between groups was compared using χ² tests.

Functional connectivity values (Fischer z-transformed) were extracted from three key clusters identified in the seed-based analysis: (1) the right mPFtha with bilateral thalamus, (2) the right Otha with the left sensory/motor cortex (precentral and postcentral gyri), and (3) the right lPFtha with the left thalamus. These clusters were selected based on their relevance to our hypotheses, as they showed significant inter-thalamic and thalamo-somatosensory connectivity, which we predicted would differentiate resilient (asymptomatic) individuals from their vulnerable (symptomatic) counterparts.

Following confirmation of normality, Pearson’s correlation coefficients were used to examine the relationships between connectivity in these clusters and psychological measures (RS-25, BDI, HTQ-total, and HTQ-PTSD subscale scores). Analyses were conducted separately for the symptomatic and asymptomatic groups to identify potential differences in connectivity patterns associated with resilience or vulnerability in trauma-exposed individuals.

To correct for multiple comparisons, we applied the Bonferroni correction within each group for correlations between connectivity values and psychological measures. With three comparisons per measure, the corrected significance threshold was set at p < 0.0167. For comparisons of correlation strength between groups, we applied the same Bonferroni-corrected threshold of p < 0.0167. All statistical analyses were performed using IBM SPSS Statistics software package (v.20).

## Results

### Descriptive statistics

The demographic characteristics, psychological measures, and clinical diagnoses for the symptomatic and asymptomatic groups are summarized in Table 1. Group differences in demographic and clinical variables were examined using independent-samples t-tests for continuous variables and a χ² test for gender distribution, which revealed no significant difference between groups (*p* = 0.753). The t-tests indicated significant differences in age, resilience (RS), and depressive symptoms (BDI). Group differences in trauma-related symptoms, as measured by both the HTQ PTSD subscale and the HTQ total score, showed trends toward significance but did not reach the threshold for statistical significance (p = 0.073 and p = 0.053, respectively). Specifically, the symptomatic group reported higher levels of depressive symptoms, while the asymptomatic group demonstrated greater psychological resilience.

**Table 1:** Demographic and Psychological Characteristics.

Variable	Symptomatic Group (n = 23)	Asymptomatic Group (n = 23)	t-value	p-vlaue
Age (years)				
Mean (SD)	30.3 (10.2)	25.2 (4.8)	2.19	0.036
Range	20 - 50	20 - 39		
Gender				
Male	16 (69.6%)	17 (73.9%)	-	-
Female	7 (30.4%)	6 (26.1%)		
RS Score				
Mean (SD)	121 (15.23)	133.30 (18.08)	−2.50	.016
Range	96 - 161	96 - 164		
BDI Score				
Mean (SD)	15.13 (6.88)	7.83 (4.05)	4.39	<.001
Range	3 - 30	1 - 16		
HTQ PTSD Score				
Mean (SD)	1.92 (0.53)	1.48 (0.35)	0.116	.073
Range	1 - 2.81	1 – 2.19		
HTQ Total Score				
Mean (SD)	1.78 (0.612)	1.39 (0.253)	0.136	.053
Range	1 – 3.60	1 – 1.80		
Diagnoses				
PTSD	5 (21.7%)	-	-	-
MDD	3 (13%)	-	-	-
Social Anxiety	2 (8.7%)	-	-	-
Panic Attacks	1 (4.3%)	-	-	-
PTSD + MDD	4 (17.4%)	-	-	-
PTSD + GAD	2 (8.7%)	-	-	-
MDD + GAD	2 (8.7%)	-	-	-
PTSD + MDD + GAD	3 (13%)	-	-	-
PTSD + MDD + Panic Attacks	1 (4.3%)	-	-	-

In terms of clinical diagnoses, all participants in the symptomatic group had at least one psychiatric diagnosis, with PTSD and comorbid PTSD and MDD being the most prevalent.

### Connectivity differences between symptomatic and asymptomatic groups

The seed-to-voxel connectivity analysis revealed significant functional connectivity differences between the symptomatic and asymptomatic groups. The contrast used (Symptomatic > Asymptomatic) identified regions where the symptomatic group demonstrated either stronger positive correlations (increased connectivity) or stronger negative correlations (decreased connectivity) compared to the asymptomatic group. Significant connectivity differences were observed in five seed regions: the right mPFtha, the left rTtha, the right Otha, and the bilateral lPFtha. Table 2 provides a detailed summary of the significant clusters, including peak coordinates and associated statistical corrected p-values for each seed region.

**Table 2:** Summary of Seed-to-Voxel Analysis Results.

Seed Region (Thalamic Subregion)	Cluster No.	Brain Region with altered connectivity	Peak Coordinates (MNI)	Cluster Size (voxels)	T-value	P-value (FDR-corrected at voxel level)	P-value (FDR-corrected across seeds)
Right mPFtha	1	LG r (Lingual Gyrus Right) OFusG r (Occipital Fusiform Gyrus Right) TOFusC r (Temporal Occipital Fusiform Cortex Right) Cereb45 r (Cerebelum 4 5 Right) Ver45 (Vermis 4 5)	+10 −56 −06	477	4.63	0.000059	0.0000885
	2	iLOC l (Lateral Occipital Cortex, inferior division Left) OFusG l (Occipital Fusiform Gyrus Left) TOFusC l (Temporal Occipital Fusiform Cortex Left)	−48 −80 −16	312	4.14	0.000165	0.0009900
	3	sLOC r (Lateral Occipital Cortex, superior division Right) OP r (Occipital Pole Right)	+22 −90 + 30	298	4.73	0.000059	0.0000885
	4	sLOC l (Lateral Occipital Cortex, superior division Left) OP l (Occipital Pole Left) Cuneal l (Cuneal Cortex Left)	−16 −88 + 26	296	4.49	0.000066	0.0000885
	5	Thalamus l Thalamus r	−08 −34 + 10	277	−6.00	0.000002	0.0000120
	6	LG l (Lingual Gyrus Left) Cereb45 l (Cerebelum 4 5 Left) Cereb6 l (Cerebelum 6 Left)	−10 −58 −08	266	4.60	0.000059	0.0000885
Left rTtha	1	PostCG r (Postcentral Gyrus Right) SPL r (Superior Parietal Lobule Right)	+14 −42 + 76	428	5.32	0.000004	0.0000120
Right Otha	1	PreCG l (Precentral Gyrus Left) PostCG l (Postcentral Gyrus Left)	−30 −18 + 70	531	4.92	0.000014	0.0000240
Right lPFtha	1	LG l (Lingual Gyrus Left) ICC l (Intracalcarine Cortex Left) Cereb45 l (Cerebelum 4 5 Left) ICC r (Intracalcarine Cortex Right)	−10 −54 −08	447	4.98	0.000011	0.0000220
	2	Thalamus l	−08 −34 + 08	268	−5.93	0.000001	0.0000120
Left lPFtha	1	LG l (Lingual Gyrus Left) Cereb45 l (Cerebelum 4 5 Left) Cereb6 l (Cerebelum 6 Left)	−10 −56 −08	316	5.69	0.000002	0.0000120
	2	LG r (Lingual Gyrus Right) Cereb45 r (Cerebelum 4 5 Right) Cereb6 r (Cerebelum 6 Right)	+14 −54 −08	288	5.55	0.000002	0.0000120

#### Right mPFtha

**Fig. 2:**
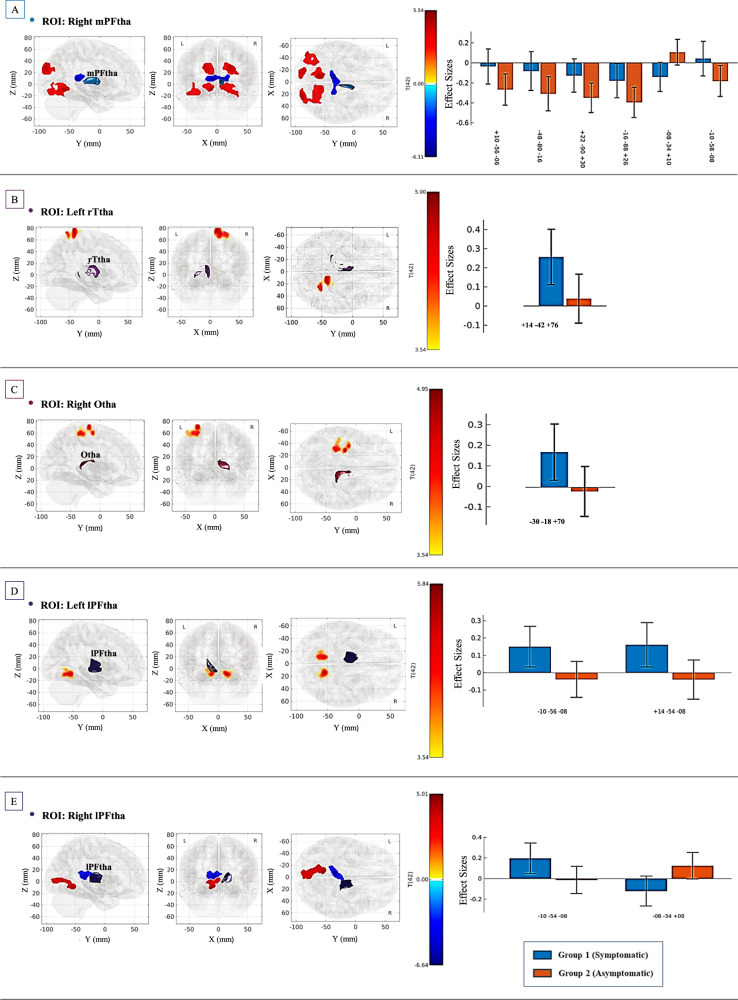
Seed-to-voxel functional connectivity analysis: significant differences between symptomatic and asymptomatic individuals. Glass displays represent the seed thalamic subregions and significant clusters of connectivity for the contrast Symptomatic > Asymptomatic. **A** The right medial prefrontal thalamus (mPFtha), showing significant connectivity with regions from visual, somatosensory, and salience networks. **B** The left rostral temporal thalamus (rTtha), demonstrating connectivity involving somatosensory and attentional networks. **C** The right occipital thalamus (Otha), highlighting connections with the somatosensory network. **D** The left lateral prefrontal thalamus (lPFtha), showing connectivity with visual, somatosensory, and salience networks. **E** The right lateral prefrontal thalamus lPFtha, demonstrating connectivity with visual, somatosensory, and salience networks. Red-yellow colours indicate regions of increased connectivity, while the blue colour represent regions of decreased connectivity. Significance is denoted by peak voxel t-statistics on the color bars, with cluster significance set at p-FDR < 0.05 and a voxel-level threshold of p < 0.001. The right panels display mean connectivity values (Fisher’s z-transformed correlation coefficients) with 90% confidence intervals for each significant cluster in symptomatic (blue) and asymptomatic (orange) groups. The x-axis represents the MNI coordinates of the identified clusters.

The right mPFtha exhibited significant functional connectivity differences with six clusters, primarily involving regions within the visual, somatosensory, and salience networks. Increased connectivity was observed between the right mPFtha and regions including the right lingual gyrus, the occipital fusiform gyrus, the temporal occipital fusiform cortex, and the cerebellum (*t* = 4.63, *p* = 0.000059). Further clusters with increased connectivity were identified between the right mPFtha and the left lateral occipital cortex, the occipital fusiform gyrus, and the temporal occipital fusiform cortex (*t* = 4.14, *p* = 0.000165); the right occipital pole and lateral occipital cortex (*t* = 4.73, *p* = 0.000059); and the left lateral occipital cortex, the occipital pole, and the cuneal cortex (*t* = 4.49, *p* = 0.000066). A significant cluster with increased connectivity was also found between the right mPFtha and the left lingual gyrus and cerebellum (*t* = 4.60, *p* = 0.000059). In contrast, decreased connectivity was observed between the right mPFtha and the bilateral thalamic regions (*t* = −6.00, *p* = 0.000002), suggesting impaired communication in the symptomatic group (Fig. 2A).

#### Left rTtha

The left rTFtha exhibited significant connectivity differences, with higher connectivity observed in the symptomatic group between the rTtha and regions within the somatosensory and dorsal attention networks, including the right postcentral gyrus and the superior parietal lobule (*t* = 5.32, *p* = 0.000004) (Fig. 2B).

#### Right otha

The right Otha exhibited increased connectivity with regions of the somatosensory network, including the precentral and postcentral gyri (*t* = 4.92, *p* = 0.000014), reflecting heightened sensory-motor reactivity in the symptomatic group (Fig. 2C).

#### Left lPFtha

The left lPFtha showed significant differences in connectivity with the visual, somatosensory, and salience networks. Specifically, the symptomatic group demonstrated increased connectivity with clusters in both hemispheres, involving the left lingual gyrus and the left cerebellum (Cereb 4/5 and Cereb 6) *(t* = 5.69, *p* = 0.000002), as well as the right lingual gyrus and the right cerebellum (Cereb 4/5 and Cereb 6) (*t* = 5.55, *p* = 0.000002) (Fig. 2D).

#### Right lPFtha

The right lPFtha exhibited significant differences in connectivity involving the visual, somatosensory, and salience networks. Increased connectivity was observed with a cluster that includes the left lingual gyrus, the intracalcarine cortex, and the cerebellum (Cereb 45 and Cereb 6) (*t* = 4.98, *p* = 0.000011). Additionally, decreased connectivity was specifically noted between the right lPFtha and the left thalamus (*t* = −5.93, *p* = 0.000001) (Fig. 2E).

### Correlations between functional connectivity and psychological measures

Group-specific correlation analyses revealed significant associations between connectivity and psychological measures in the symptomatic group, particularly involving the right Otha and the right lPFtha.

For the right Otha, connectivity between this seed and a cluster in the somatosensory network (the left precentral and postcentral gyri) demonstrated a significant negative correlation with RS-25 scores in the symptomatic group (r = −0.506, p = 0.014), surviving the Bonferroni correction threshold (p < 0.0167). This negative association suggests that a higher connectivity between the right Otha and the somatosensory regions was linked to lower resilience. For the right lPFtha, connectivity with the left thalamus was also found to negatively correlate with resilience in the symptomatic group (r = −0.506, p = 0.014), meeting the Bonferroni-corrected significance threshold. This finding indicates that reduced connectivity between the right lPFtha and the left thalamus was associated with lower resilience levels.

While a positive correlation between functional connectivity from the right lateral prefrontal thalamus (lPFtha) to the left thalamus and depressive symptoms (BDI scores) was observed in the symptomatic group (r = 0.463, p = 0.026), this effect did not survive Bonferroni correction (p > 0.0167). No significant correlations with resilience or depressive symptoms were observed in the asymptomatic group after correction (all p > 0.0167).

**Fig. 3:**
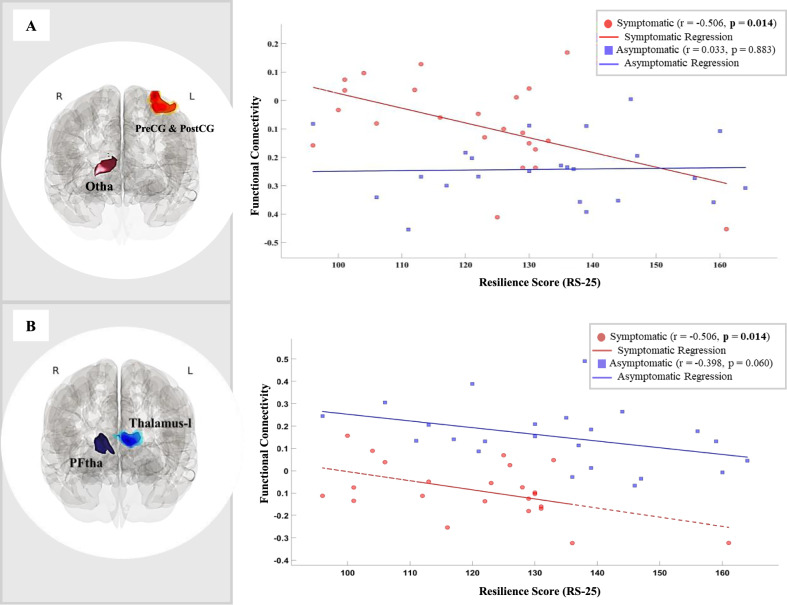
Group differences in the association between functional connectivity and resilience scores (RS-25). Scatter plots illustrate the relationship between functional connectivity and resilience scores (RS-25) in symptomatic (red) and asymptomatic (blue) individuals, with linear regression lines fitted for each group. **A** A significant negative correlation was observed in symptomatic individuals between functional connectivity of the right occipital thalamus (Otha) and the precentral and postcentral gyri and resilience scores (r = −0.506, p = 0.014), suggesting that increased connectivity in this region is associated with lower resilience. No significant correlation was found in asymptomatic individuals (r = 0.033, p = 0.883). **B** A significant negative correlation was found between functional connectivity of the right lateral prefrontal thalamus (lPFtha) and the left thalamus and resilience scores in symptomatic individuals (r = −0.506, p = 0.014), indicating that impaired thalamic connectivity may be linked to reduced resilience. In asymptomatic individuals, a trend-level negative correlation was observed (r = −0.398, p = 0.060). Statistical significance was determined using a corrected p-value threshold of 00.0167.

Scatter plots illustrating the significant correlations between connectivity and RS-25 scores for the symptomatic group are presented in Fig. 3.

## Discussion

The aim of our study was to reveal the neurobiological mechanisms that underlie both resilience and vulnerability to trauma by examining thalamic subregions connectivity patterns in refugees exposed to prolonged and severe trauma. Using UHF rs-fMRI, we compared the functional connectivity of thalamic subregions between those who developed psychopathology (symptomatic) and their resilient (asymptomatic) counterparts.

Our findings revealed significant differences in thalamic connectivity, highlighting altered communication across the thalamic subregions. Resilient individuals exhibited increased inter-thalamic connectivity, potentially reflecting more efficient sensory integration and emotion regulation, while symptomatic individuals showed disrupted connectivity, likely contributing to emotional dysregulation and heightened reactivity to sensory stimuli.

Additionally, symptomatic individuals demonstrated increased connectivity between the thalamic subregions and the somatosensory and motor networks, which likely drives hyperarousal and heightened sensory reactivity. In contrast, resilient individuals displayed reduced connectivity with these networks, possibly serving as an adaptive mechanism to mitigate the psychological impact of trauma.

Thus, our observations shed light on the unique contribution of each thalamic subregion in mediating resilience or vulnerability to trauma, offering deeper insights into the neurobiological mechanisms underlying these adaptive or maladaptive responses.

This pattern highlights that resilience may be supported by more effective thalamic integration and reduced sensory interference.

These group differences in thalamic connectivity can be contextualized within broader neurobiological models of trauma and resilience. The thalamus is central to sensory integration, salience detection, and the regulation of arousal through its reciprocal connections with prefrontal, limbic, and sensory cortices [21, 22, 61]. Neurobiological models suggest that resilience is supported by effective thalamocortical gating, where sensory inputs are adaptively filtered, allowing for emotional regulation and attentional control in the face of stress [62, 63]. Heightened connectivity in the symptomatic group may reflect disrupted gating or compensatory over-recruitment, as seen in trauma-related hypervigilance and sensory overload [25, 64, 65]. In contrast, the increased inter-thalamic connectivity and reduced connectivity with sensory regions observed in asymptomatic individuals may reflect more efficient thalamic modulation of bottom-up input, in line with models of adaptive neural inhibition and emotional resilience. These findings suggest that thalamic circuits play a pivotal role in modulating vulnerability versus resilience by balancing the flow of affective and sensory information across the brain.

### Right mPFtha: integrating sensory, emotional, and salience networks

The right mPFtha plays a key role in integrating sensory and emotional information and is critically involved in emotion regulation and decision-making. This thalamic subregion maintains reciprocal connections with areas of the prefrontal cortex, which makes it essential for cognitive control, executive functioning, and emotional modulation, reflecting its broader involvement within the dorsomedial thalamus [16].

The decreased inter-thalamic connectivity seen in the symptomatic group suggests that the impaired communication between the thalamic regions likely contributes to emotional dysregulation, a core feature of trauma-related psychopathology. In contrast, increased functional connectivity was observed between the right mPFtha and regions in the visual network (lingual gyrus, occipital fusiform gyrus, occipital pole, and cuneal cortex), the somatosensory network (lateral occipital cortex), and specific cerebellar regions (Cereb 6, Cereb 45, and Vermis 4/5). This pattern of hyperconnectivity suggests that symptomatic individuals may experience heightened emotional reactivity and hypervigilance in response to sensory stimuli, consistent with the findings involving PTSD, MDD, and GAD [26, 66, 67]. Specifically, hyperconnectivity within the visual network, particularly involving areas such as the occipital cortex and the fusiform gyrus, has been linked to increased visual excitability and sensory overload, contributing to heightened emotional reactivity in MDD and GAD [68, 69]. Recent evidence suggests that an altered connectivity within the visual cortex may disturb the balance between excitation and inhibition, leading to heightened emotional and sensory responses, thus making it more challenging for individuals to disengage from distressing stimuli. Such dysfunction may perpetuate symptoms like hyperarousal and emotional dysregulation [70].

The identified cerebellar regions are associated with both the somatosensory and salience networks. Cerebellar regions 4/5 (Cereb 45) and Vermis 4/5 are linked to the somatosensory network, contributing to sensory integration and motor coordination, while Cerebellum 6 (Cereb 6) is part of the salience network, which plays a critical role in attention allocation and the processing of emotionally salient stimuli [71, 72]. In the symptomatic group, the increased connectivity between the right mPFtha and these cerebellar regions may indicate an overactive response to emotionally relevant stimuli, contributing to difficulties in emotion regulation. Hyperconnectivity involving the cerebellum has also been reported in individuals with MDD and PTSD, highlighting its role in exaggerated attentional and emotional responses that contribute to trauma-related psychopathology [26].

In contrast, the asymptomatic group demonstrated increased inter-thalamic connectivity alongside reduced connectivity between the mPFtha and the sensory and visual networks. This pattern likely reflects a more efficient integration of sensory and emotional information, allowing these individuals to effectively regulate their responses to external stimuli, thereby fostering resilience. Such efficient inter-thalamic communication appears to facilitate a better management of sensory inputs, preventing emotional overwhelm and supporting stability even when exposed to trauma.

### The lPFtha: complementing emotional and sensory regulation

The lateral prefrontal thalamus (lPFtha) is involved in both sensory processing and emotion regulation, akin to the dorsomedial nucleus, which has connections to the prefrontal and cingulate cortices that support cognitive control and emotion modulation [16]. The left and right lPFtha subregions demonstrated connectivity patterns that mirrored findings from the right mPFtha, specifically involving the visual (lingual gyrus, intracalcarine cortex), somatosensory (Cereb 45), and salience (Cereb 6) networks in both the symptomatic and asymptomatic groups.

In the symptomatic group, heightened connectivity between the lPFtha and these networks reflects an increased sensitivity to emotionally salient stimuli, alongside difficulties in integrating sensory information, paralleling the findings from the mPFtha. This pattern supports the notion that symptomatic individuals may struggle with sensory overload and emotional reactivity, underscoring the role of these thalamic subregions in the emotional dysregulation linked to trauma-related psychopathologies [26, 66, 67, 70]. Conversely, the reduced connectivity between the lPFtha and these networks seen in the asymptomatic group confirms a more efficient integration of sensory and emotional information, enabling these individuals to regulate their responses to external stimuli more effectively, thus fostering resilience.

Additionally, the decreased connectivity between the right lPFtha and the left thalamus seen in the symptomatic group negatively correlated with the group’s resilience scores, indicating that an impaired thalamic communication is associated with lower resilience. In the asymptomatic group, however, no significant correlations were found. While this may suggest that more balanced thalamic connectivity supports emotional stability and resilience, it is also possible that the absence of associations reflects a restricted range in resilience scores within this group (mean = 133.30, SD = 18.08; range = 96–164), which may have limited the sensitivity to detect statistical effects.

Overall, the findings from the lPFtha complement those from the mPFtha, underscoring the importance of coordinated thalamic connectivity for the maintenance of emotional stability. The shared connectivity patterns involving the visual, somatosensory, and salience networks prove that resilience is linked to efficient sensory filtering and emotion regulation, whereas vulnerability is characterized by hyperconnectivity and dysregulation across these critical thalamic-cortical circuits.

### Right otha: heightened sensory and motor reactivity

The right Otha is a subregion primarily involved in relaying visual and somatosensory information to the cortex, contributing significantly to both sensory perception and motor control. The role of this subregion aligns with the pulvinar nucleus of the thalamus, which is critical for processing visual information and facilitating multisensory integration, functions that are particularly relevant for modulating attention and guiding motor responses [16].

In the symptomatic group, the increased connectivity between the Otha and regions in the somatosensory network, particularly the precentral and postcentral gyri, may indicate heightened sensory reactivity, a hallmark frequently observed in trauma-related psychopathology. This pattern is consistent with prior findings in PTSD, where hyperactivity in sensorimotor areas has been associated with hyperarousal and difficulties in regulating responses to environmental stimuli [73]. Alternatively, the observed hyperconnectivity could reflect a compensatory mechanism, whereby the brain attempts to modulate or adapt to increased sensory input following trauma exposure. Importantly, as resting-state functional connectivity reflects statistical dependencies rather than directed causal relationships, interpretations regarding the functional significance or directionality of these findings remain inferential and should be approached with caution. Our recent findings in patients with major depressive disorder (MDD) also revealed hyperconnectivity between the thalamus and sensorimotor cortices, suggesting that this altered connectivity pattern may be shared across different trauma-related conditions [74]. This aligns with clinical observations that PTSD patients frequently experience sensory filtering deficits, including heightened stimulus sensitivity and sensory flooding [38], which may contribute to persistent hypervigilance and emotional dysregulation. Further support for this interpretation comes from the correlation analyses. In the symptomatic group, the connectivity between the right Otha and the somatosensory network (left precentral and postcentral gyri) was found to be negatively correlated with resilience scores, suggesting that increased connectivity in these regions may reflect sensory overload and heightened emotional arousal.

In contrast, no significant correlations were observed in the asymptomatic group. While this may reflect a more adaptive neural mechanism that allows asymptomatic individuals to manage sensory input more effectively, the absence of significant correlations could also be partly due to the reduced variability in resilience scores in this group, potentially limiting statistical power [75].

### Left rTtha: sensory integration and attentional control

The left rTtha, which is crucial for sensory integration and attentional control, exhibited significant functional connectivity differences between the symptomatic and asymptomatic groups. Functionally, this subregion aligns with the ventral lateral and anterior nuclei of the thalamus, which are involved in coordinating sensory input with attentional mechanisms, ultimately aiding in efficient information processing and decision-making [16].

In the symptomatic group, the increased connectivity between the rTtha and regions within the somatosensory network (postcentral gyrus) and the dorsal attention network (superior parietal lobule) suggests impaired attentional control. This heightened connectivity is indicative of a reduced capacity to filter out irrelevant stimuli, potentially leading to sensory overload and an inability to regulate attention effectively. Such dysfunctions may contribute to hypervigilance, which is commonly observed in trauma-related disorders like PTSD and MDD [73, 74].

Moreover, the increased connectivity observed between the rTtha and the dorsal attention network (particularly, the superior parietal lobule) further highlights the difficulties in regulating attentional focus and filtering sensory inputs. This is consistent with the findings from studies involving panic and social anxiety disorders, which show similar hyperactivation in attentional networks, exacerbating symptoms of anxiety and impaired attentional control [76–78]. Specifically, the heightened connectivity of the dorsal attention network can impair an individual’s ability to focus attention appropriately, thus perpetuating hypervigilance and emotional dysregulation [78].

In contrast, the asymptomatic group exhibited more balanced connectivity in these regions. The reduced connectivity seen between the left rTtha and regions involved in sensory processing and attention control suggests that adaptive sensory integration and attentional regulation are possible protective factors against trauma-related psychopathology. This adaptive capacity allows resilient individuals to manage environmental stressors without overwhelming emotional responses, promoting psychological stability and reducing vulnerability.

These results complement the findings from the right Otha, where increased connectivity with the somatosensory regions was found to be similarly associated with heightened sensory reactivity in symptomatic individuals. Together, these findings emphasize the broader role of the thalamic subregions in coordinating sensory and attentional functions, suggesting that disruptions in thalamic connectivity may underpin sensory overload and attentional dysregulation, both being hallmarks of trauma-related psychopathology. The integration of these multiple connectivity patterns highlights the thalamus’s pivotal role in maintaining balanced sensory and emotional processing to support resilience.

### Integrated connectivity findings across thalamic subregions

The thalamic subdivisions, including the mPFtha, lPFtha, and Otha, revealed consistent patterns of dysregulated connectivity, differentiating symptomatic individuals from their resilient counterparts. In symptomatic individuals, hyperconnectivity with the visual, somatosensory, and salience networks across multiple thalamic subregions features a shared mechanism of vulnerability, which is characterized by heightened sensitivity to emotionally salient stimuli and impaired sensory integration, likely contributing to emotional dysregulation, sensory overload, and hypervigilance, which are the core features of trauma-related disorders such as PTSD and MDD [15, 66, 79].

Conversely, the asymptomatic group exhibited consistently higher levels of inter-thalamic connectivity and reduced engagement with the sensory networks. This connectivity pattern suggests a resilience mechanism involving efficient sensory filtering and emotion regulation, which allows these individuals to maintain emotional stability despite trauma exposure [70, 80]. These results emphasize the role of effective thalamic communication in adaptive coping, pointing to inter-thalamic connectivity as a potential neural marker of resilience.

Importantly, the involvement of the visual, somatosensory, and salience networks across these thalamic subregions highlights the thalamus’s central role as an integrative hub for the modulation of sensory and emotional responses. A disrupted connectivity between the thalamus and these cortical networks may serve as a transdiagnostic marker of vulnerability across different psychiatric disorders, while an enhanced inter-thalamic connectivity may represent a resilience-promoting mechanism mitigating the impact of traumatic stress.

## Conclusion and future directions

This study underscores the crucial role of thalamic connectivity in shaping trauma responses, particularly in distinguishing resilience from vulnerability among trauma-exposed individuals. Using ultra-high field 7 T fMRI, we identified distinct thalamic networks involved in emotion regulation, sensory processing, and attentional control. The disrupted inter-thalamic connectivity and increased connectivity with the sensory and motor regions seen in individuals with trauma-related psychopathology likely contributed to the hypervigilance and emotional dysregulation in these individuals. Conversely, resilient individuals exhibited more regulated connectivity patterns, which support emotional stability and efficient sensory integration. These findings align with the existing literature linking thalamic connectivity to PTSD and MDD, while offering new insights into the neurobiological mechanisms that underlie resilience.

The observed correlation between reduced inter-thalamic connectivity and lower resilience in the symptomatic group underscores the key role of thalamic communication in the maintenance of emotional stability. Although no significant correlations between connectivity and symptom severity were identified, the strong link between connectivity and resilience highlights the potential for interventions targeting these neural pathways to enhance resilience in trauma-exposed populations. While asymptomatic status may reflect an absence of diagnosable psychiatric symptoms, resilience was assessed separately using the RS-25 as a dimensional construct. This distinction allowed us to examine adaptive functioning beyond clinical diagnosis.

Clinically, these findings suggest promising directions for therapeutic interventions. Targeted approaches that modulate thalamic connectivity may improve emotion regulation and resilience in trauma-exposed individuals. Interventions such as neurofeedback, transcranial magnetic stimulation (TMS), and eye movement desensitization and reprocessing (EMDR) have shown potential in influencing thalamocortical circuits implicated in affective regulation. Neurofeedback may enhance thalamic modulation indirectly by training individuals to regulate attentional and arousal networks, though findings remain mixed and warrant further replication [81, 82]. TMS, especially when targeting the dorsolateral or medial prefrontal cortex, has demonstrated the capacity to influence downstream thalamic activity via corticothalamic projections [83]. EMDR, which has demonstrated clinical efficacy in trauma treatment, is proposed to facilitate adaptive information processing and multisensory integration, mechanisms likely involving thalamic pathways [84, 85]. Such interventions, aimed at normalizing thalamic connectivity, may enhance emotion regulation and sensory integration, thereby providing valuable therapeutic benefits for trauma-related psychopathology [82, 85]. Given the heightened vulnerability of refugee populations to trauma-related disorders, these findings are especially relevant for developing interventions to help enhance resilience in this high-risk group. By targeting the thalamic circuits involved in emotion regulation and sensory processing, we may enhance emotional stability and coping mechanisms in individuals with a prolonged experience of trauma exposure.

Future research should focus on longitudinal studies to explore the evolution of thalamic networks over time, particularly regarding their adaptation in response to ongoing trauma and therapeutic interventions. Understanding how specific therapies, such as neurofeedback, TMS, EMDR, and cognitive-behavioral therapy (CBT), affect thalamic connectivity can offer further insights into how these neural pathways can be modulated to foster resilience. This approach may help identify which therapeutic techniques are most effective in promoting recovery and resilience among trauma-affected populations. Moreover, expanding this line of research to include diverse trauma-exposed populations would contribute to a broader understanding of resilience mechanisms and their potential modulation across different contexts.

## Limitations

We acknowledge several limitations that may affect the interpretation of our findings. Although we did not quantify trauma duration or time since displacement, all participants were exposed to war-related trauma due to the prolonged conflict in the Levant region. This likely resulted in a relatively consistent pattern of chronic exposure across the sample, though individual variability cannot be excluded. While age was statistically controlled in all second-level analyses, developmental or age-related factors may still influence neural functioning and resilience mechanisms in trauma-exposed individuals, and these aspects were not specifically examined. The cross-sectional design of the study limits conclusions regarding causality and the direction of associations between brain connectivity and psychiatric status. Additionally, although we employed validated self-report instruments (BDI, HTQ, RS-25), such tools may be subject to recall bias and subjective interpretation. Finally, participants diagnosed with PTSD, MDD, or anxiety disorders were grouped together into a single symptomatic category. While this may limit diagnostic specificity, it reflects the high comorbidity among trauma-related conditions and the practical challenges of recruiting diagnostically homogeneous samples in refugee populations.

## Data Availability

Source data can be made available through contact with the principal investigator, Prof. Dr. Neuner.
